# Jabuticaba (*Plinia* sp.) Peel as a Source of Pectin: Characterization and Effect of Different Extraction Methods

**DOI:** 10.3390/foods12010117

**Published:** 2022-12-26

**Authors:** Laís M. Resende, Adriana S. Franca

**Affiliations:** 1 PPGCA, Universidade Federal de Minas Gerais, Av. Antônio Carlos, 6627, Belo Horizonte 31270-901, Brazil; 2 DEMEC, Universidade Federal de Minas Gerais, Av. Antônio Carlos, 6627, Belo Horizonte 31270-901, Brazil

**Keywords:** jabuticaba, pectin, ultrasound extraction, microwave extraction, cellulase, hemicellulase, antioxidants, FTIR, agricultural waste valorization

## Abstract

The peel of jabuticaba, a small fruit native to Brazil, has been shown to be a potential source of antioxidants and soluble dietary fibers. In this study, flours prepared from these peels were evaluated as a source of pectin. Different extraction methods were employed: ultrasound (US) extraction followed by low temperature heating (40 °C); in a microwave (MW) without (method 1) or with cellulase (method 2) or hemicellulase (method 3); or in a water bath (method 4). Pectin yields ranged from approximately 18% for methods 1 and 4 up to 22% for enzyme-assisted extractions (methods 2 and 3). Methods that did not employ enzymes resulted in low amounts of methoxyl pectins, as opposed to high amounts of methoxyl pectins obtained after enzyme treatment. Cyanidin-3-*O*-glucoside (C3G) and ellagic acid were the main phenolic compounds found in jabuticaba peel pectins, with higher C3G levels obtained with enzyme-free extraction (methods 1 and 4). All pectins from jabuticaba peel presented a reddish tone, good emulsifying properties and high swelling capacity. The pectin extracted using US+MW+cellulase (method 2) presented better emulsifying performance (higher values of emulsifying activity and emulsion stability), more effective than commercially available citrus pectin.

## 1. Introduction

Jabuticaba (*Plinia cauliflora*) is a small fruit native to Brazil. Its sweet pulp is used in the preparation of beverages, jams and desserts. Its dark purple or black peel corresponds to about 40% of its mass and can be viewed as a source of anthocyanins and dietary fibers. The soluble dietary fiber content has been reported to range from approximately 4 to 9 g/100 g of dry peel [1,2].

Pectins are soluble dietary fibers used in the food industry as gelling and stabilizing agents, emulsifiers, viscosity enhancers, thickeners and texture modifiers. Pectins are polysaccharides that occur in the cell walls of plant tissues in three polymeric forms: (i) homogalacturonan (HG), a linear polymer of α-(1-4)-linked galacturonic acid (GalA); (ii) rhamnogalacturonan I (RG-I), a repeating disaccharide of α-1,2-linked-L-rhamnose-α-1,4-D-GalA with various side chains of L-rhamnosyl which can be substituted with neutral sugar and glucuronic acid; and (iii) rhamnogalacturonan II (RG-II), a HG backbone with different monosaccharides side chains attached. Residues of GalA in HG can be methyl-esterified and/or *O*-acetyl-esterified. The degree of methyl-esterification (DM) interferes with the technological properties of pectins [3,4,5]. High methoxyl pectins (HMP) are pectins that contain DM levels above 50% and form gels at high concentrations of sugar (55–75%) in acidic systems (pH 2.5–3.5), while low methoxyl pectins (LMP) (DM < 50%) form gels in the presence of divalent ions in a wide pH range (2–6).

Commercial pectin is obtained mainly from citrus peel and apple pomace, but nonconventional sources have been also investigated due to the interest in pectins with different functional properties [6]. Different residues of fruit and vegetable processing have been shown as potential sources of pectin, including tomato, carrot, olive and grape pomaces and mango, passion fruit, banana and pequi peels [4,7]. Jabuticaba jams and beverages have a natural thickening effect attributed to the polysaccharide composition of their peels [8], thus pointing at jabuticaba peel as potential sources of pectin. Benvenutti, Sanchez-Camargo, Zielinski and Ferreira [9] investigated this potential by extracting pectin from jabuticaba pomace, and obtained yields ranging from 4 to 27%, depending on the employed solvent. Nascimento et al. [10] also evaluated the potential of jabuticaba fruit commercial flour as a pectin source, and obtained a product with 95% GalA that was shown to have an anticancer effect. These authors compared pectins extracted from plum, papaya and jabuticaba, with the latter presenting better results regarding the inhibition of in vitro growth of colon cancer cells. Such action was attributed to the higher concentration of GalA observed in the jabuticaba pectin. Nonetheless, it is noteworthy to point out that coextracted antioxidants and phenolics may also have contributed to the anticancer effect. Both studies confirmed the potential of jabuticaba and its pomace as a source of pectin. However, neither the influence of the extraction temperature nor the use of non-conventional extraction techniques for pectin were investigated.

The yield, structure and consequently the physicochemical properties of pectins depend on the source and the conditions of extraction [3,4,5,6,7], with low pH water acidified with mineral acids and high temperatures usually being employed. However, the use of organic acids, such as citric acid, has intensified recently as they are more interesting from an environmental point of view. They also provide higher yields, due their lower hydrolyzing capacity, which results in less degraded and longer pectin chains [4,5]. Low pHs avoid the extraction of pectins from cell walls in their original structures, as hydrolysis and beta-elimination reactions with temperatures above 23°C are favored. The use of lower temperatures (25 and 40°C) has also been considered and, although it has showed lower yields and lower GalA proportion, more intact pectins (whose structures are more similar to that found in the plant cell wall) are produced, with higher apparent viscosity. They were also shown to present more effective biological activities with respect to galectin-3, a cancer-associated protein [11].

The conventional extraction of pectins is associated with large consumption of time, energy and solvents, in addition to being thermally unsafe. Innovative extraction approaches like ultrasound (US), microwave (MW) and enzyme have been investigated to overcome the aforementioned limitations, in order to increase yield and improve pectin characteristics [5,12]. Ultrasound-assisted extraction (UAE) is a non-thermal technique, based on sonication to propagate sound waves that create cavitations in the liquid. The collapse of cavitation bubbles near plant material causes an increase in pressure and temperature, which results in cell disruption. Microwave-assisted extraction (MAE) is a technique that involves short extraction times, based on internal heating by microwaves, with a consequent increase in pressure and tissue disruption and release of compounds into the solution.

Enzyme-assisted extraction (EAE) is a method based on the disintegration of the cell wall by specific enzymes such as cellulases and hemicellulases, increasing both the yield and quality of pectins [5,12,13]. Cellulase is a multiple enzyme system comprised of endo- and exo-1,4-β-D-glucanases and cellobiase (β-D-glucano hydrolase). Endo-glucanase hydrolyzes carboxyl methyl cellulose, resulting in a rapid decrease in chain length and a slow increase in reducing groups. It also acts on cellodextrins, the intermediate products of cellulose hydrolysis, converting them to cellobiose and glucose. Exo-glucanase degrades cellulose by removing the cellobiose units from the non-reducing end of the chain. Cellobiase completes the process by cleaving cellobiose and removing glucose from the non-reducing ends of oligosaccharides. Thus, the complete degradation of cellulose to glucose requires the synergistic action of all three components of the enzyme system.

The hemicellulose substrate is a complex carbohydrate structure consisting of different easily hydrolysable polymers including pentoses (xylose and arabinose), hexoses (mannose, glucose and galactose) and sugar acids. Thus, its variable structure and organization requires the combined action of many enzymes for its complete degradation. In this regard, hemicellulases share similar activities with cellulases, because of the common β-1,4-glycosidic bonds in the backbone of the hemicellulose. One of the main catalytic modules of hemicellulases is glycoside hydrolases (GHs) that hydrolyze glycosidic bonds. Another main catalytic module of hemicellulases is carbohydrate esterase, which hydrolyzes ester linkages of acetate or ferulic acid side groups. Xylanases, α-d-glucuronidases, α-l-arabinofuranosidases, α-d-galactosidases, β-xylosidases and β-mannanases attack glycosidic bonds, whereas acetyl or feruloyl esterases hydrolyze ester bonds of acetate or ferulic acid side groups in the plant cell wall structure.

The previously described methods can be also combined to improve extraction efficiency and product quality. Liew et al. [12] employed sequential ultrasound–microwave-assisted extraction (UMAE) to extract pectins from pomelo peels. The combination of techniques resulted in increases in yield and GalA content, as well as improvements in morphological characteristics in comparison to each individual technique. Ultrasound–enzyme-assisted extraction (UEAE) is a combined technique that has been also shown to provide increases in yield and higher values of elastic modulus for pectin-enriched fractions obtained from discarded carrots [14]. Pectin extraction methods combining microwaves and enzymes have been found in the literature. Since extraction by enzymes is performed at low temperatures, it is possible to use both methods simultaneously, and thus explore their synergism. Furthermore, both methods can be used sequentially with ultrasound extraction.

In view of the aforementioned studies, the objectives of this work were to compare the effect of different extraction methods on the yield and characteristics of pectins obtained from jabuticaba peels. In this study we evaluated for the first time the potential of combining UMAE with EAE, namely ultrasound–microwave–enzyme-assisted extraction (UMEAE), for pectin extraction.

## 2. Materials and Methods

### 2.1. Materials

The jabuticaba peel flour used in this study was produced from ripe fruits obtained from the municipality of Entre Rios de Minas, located in the state of Minas Gerais, Brazil. The flours were produced by drying the fruit peels in a convective oven at 60 °C for 20 h, followed by grinding, sieving (425 μm) and storage at −18 °C. The prepared flours contained 11.25 g/100 of moisture, 6.50 g/100 g of soluble dietary fiber and 845 mg/100 g and 13 g gallic acid equivalents (GAE)/100 g of total monomeric anthocyanins and total extractable phenolics, respectively [2].

HLPC grade reagents included standards of monosaccharides and the following antioxidants: cyanidin-3-*O*-glucoside and ellagic acid. The employed enzymes were cellulase solution from *Trichoderma reesei* (Sigma-Aldrich ATCC 26921, enzymatic activity ≥ 700) and hemicellulase from *Aspegillus niger* (Sigma-Aldrich H2125-150KU). Hemicellulase activity: one unit will produce a relative fluidity change of 1 per 5 min using locust bean gum as substrate at pH 4.5 at 40 °C. Cellulase activity: one unit will liberate 1.0 micromole of D-glucose from cellulose per hour at pH 5.0 at 37 °C (2 hour assay). The chemicals for chromatographic analysis were trifluoroacetic acid, sodium borohydride, dimethylsulfoxide, 1-methylimidazole, dichloromethane, ethanol, methanol and acetic acid, all purchased from Sigma-Aldrich (São Paulo, Brazil). The other employed chemicals were of analytical grade and acquired from Neon Comercial Reagentes Analíticos (São Paulo, Brazil) and Labsynth Produtos para Laboratórios (São Paulo, Brazil). Commercial citrus pectin (CCP, DM = 75.7%) was obtained from Dinâmica Química Contemporânea (São Paulo, Brazil).

### 2.2. Pectin Extraction

**Figure 1:**
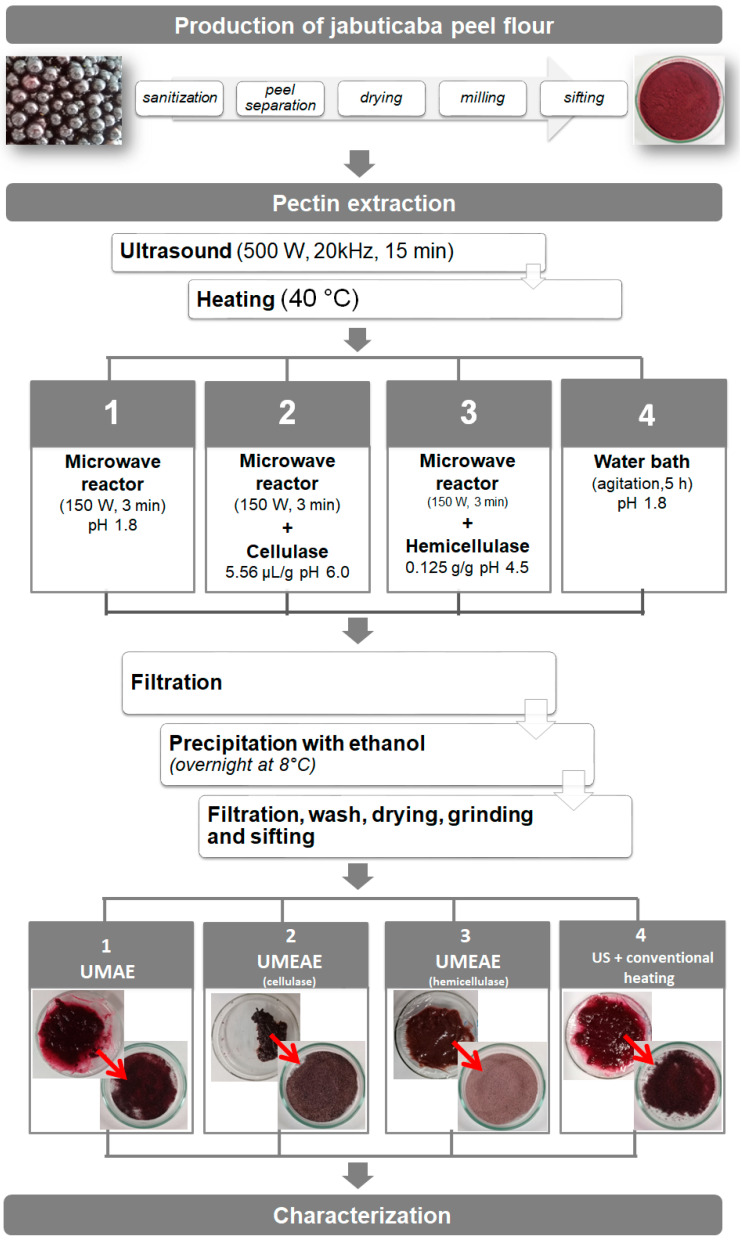
Flowchart of the production of pectins from jabuticaba peels.

The procedure developed for evaluation of the different pectin extraction methods, based on the works of Chen et al. [11], Liew et al. [12], Encalada et al. [14], Dominiak et al. [15] and Leão et al. [16], is summarized in Figure 1. The jabuticaba peel flour (5g) was mixed with 145 mL of the specific solution (see description according to the employed method) in triplicate. The mixture was then transferred to a 200 mL beaker and sonicated continuously in an ultrasonic reactor (EcoSonics, 500 W, 20 kHz, standard 1/2” diameter probe placed at 50 mm from the vessel bottom) for 15 min [11]. The solution was transferred to a 500mL flat-bottomed flask prior to microwave heating and the differences in extraction methods are detailed as follows. Method 1 (UMAE) consisted of using citric acid solution (pH 1.8) and heating in a microwave irradiated unit (Model StartSynth, Milestone, Italy) equipped with a reflux condenser, a magnetic stirrer bar and an infrared feedback temperature control system at 150 W for 3 min. For method 2 (UMEAE), a citrate buffer solution was employed (pH 6.0) with 280 µL of cellulase solution, and microwave heating was performed as already described for method 1. Method 3 (UMEAE) employed citrate buffer solution (pH 6.0) and 0.625 g of hemicellulase with microwave heating as previously described. For method 4 (US + conventional heating) the flour was mixed with citric acid solution (pH 1.8) in a 250mL beaker and then heated in a Dubnoff water bath with temperature control and constant agitation (100 rpm) for 5 h. Afterwards, the mixtures were filtered and the pectins were precipitated with 95% (*v*/*v*) ethanol (2 volumes of ethanol per volume of filtrate, 12 h at 8 °C), filtered through a stainless steel sieve (250 μm), washed with ethanol 70 % (*v*/*v*), dried (40 °C, 12 h), ground and sieved (425 μm), following the procedure described by Leão et al. [16].

The yield was calculated as follows:

yield (%) = m_1_/m_2_ × 100 (1)

where m_1_ is the mass of dried pectin (g) and m_2_ is the mass of dried jabuticaba peel flour (g). The insoluble components remaining after pectin extraction were also dried, ground and sieved as previously described, and used for determination of monosaccharide content [11].

### 2.3. Pectin Characterization

#### 2.3.1. FTIR Analysis and DM

FTIR analyses were performed using a Shimadzu IRAffinity-1 FTIR Spectrophotometer (Japan) with a deuterated L-alanine-doped triglycine sulfate detector. An ATR sampling accessory (MIRacle) with ZnSe windows was employed. Spectra were registered in the 4000–600 cm^−1^ range with 4 cm^−1^ resolution and 20 scans in a dry atmosphere at 20 ± 0.5 °C [2]. Spectral data were subjected to baseline correction using the IRsolution Software Ver.1.20 (Shimadzu Corporation, Japan). The DM was estimated by FTIR using the bands at 1630 and 1740 cm^−1^ and Equation 2, as proposed by Liew et al. [12] and Kyomugasho, Christiaens, Shpigelman, Loey and Hendrickx [17].

DM (%) = *A*_1740_/(*A*_1740_ + *A*_1630_) × 100 (2)

where *A*_1740_ corresponds to the absorbance at 1740 cm^−1^ (methyl-esterified carboxyl groups) and *A*_1630_ corresponds to the absorbance at 1630 cm^−1^ (non methyl-esterified carboxyl groups).

#### 2.3.2. Determination of Neutral Monosaccharides

The neutral monosaccharide composition was evaluated in the pectins and their extraction residues using the alditol acetate derivatization method by gas chromatography, as described by Resende, Franca and Oliveira [18]. Briefly, the neutral sugars present in the samples were hydrolyzed to their alditol acetates with trifluoroacetic acid, reduced with sodium borohydride in dimethyl sulfoxide and derivatized with acetic anhydride in the presence of 1-methylimidazole. Dichloromethane was used to extract the alditol acetates, the separation of which was performed in a Varian 3900 gas chromatograph with flame ionization detector using a BPX-70 capillary column (30 m × 0.32 mm × 0.25 μm) and nitrogen as the carrier gas (1.5 mL/min). The injector temperature was 230 °C and the detector temperature was 280 °C. The duration of the run was 38 min and determinations were performed in duplicate. Neutral sugars were identified by comparison with standards. Allose was used as an internal standard.

#### 2.3.3. Phenolic Compounds

Phenolic compounds were extracted according to Barros et al. [19] with adaptations. Briefly, the flour (0.1 g) was mixed with 5 mL of ethanol 50% (*v*/*v*) and acetic acid (final pH 3.0 ± 0.1) in tubes protected from light and sonicated in an ultrasonic bath for 60 min at 30 °C. After this process, the extracts were centrifuged at 3500 rpm for 5 min and the remaining material was mixed with ethanol 50% (*v*/*v*) and centrifuged for 5 min. The supernatants were combined and the pH was adjusted to 3.0 where necessary.

The content of phenolic compounds was determined by high-performance liquid chromatography (HPLC), as described by Plaza et al. [20] with adaptations. The extracts were diluted with ultrapure water (final concentration of 0.0025 g/mL) and filtered through a syringe filter (0.22 µm). The analysis was performed using a prominence model chromatograph (Shimadzu, Japan) with DAD detector and reverse-phase column C18 (5 µm particle size, 4.6 µm × 150 mm) at 30 °C. The injection volume was 20 μL and the mobile phases consisted of aqueous formic acid 0.5% (*v/v*) (solvent A) and 0.5% (*v*/*v*) of formic acid in methanol (solvent B). The elution gradients were 0 min, 5% (B); 0–5 min, 5% (B); 5–45 min, 50% (B); and 45–53 min, 50% (B). The flow rate was 0.6 mL/min. The UV–Vis detector collected the signal at 520 nm for C3G and 350 nm for ellagic acid, which were identified by comparison with the retention time and spectrum of authentic standards and used for the construction of calibration curves.

#### 2.3.4. Technological Properties

Color parameters L* (luminosity), a* and b* were measured by a tristimulus colorimeter (ColorFlex, Hunter Associates Laboratory, VA) using 10° observer angle and the D65 illuminant. Polar coordinates c* (chroma) and h° (hue angle) were calculated as described by Resende et al. [2].

The water solubility index (WSI), swelling capacity (SC) and water and oil retention capacities (WRC and ORC) were evaluated according to Resende et al. [18], with a slight modification in the mass used in the WRC and ORC tests. Briefly, 0.1 g of pectin sample was mixed with 2 mL of water (for WRC) or oil (for ORC) and the mixture was subjected to agitation for 30 min and subsequent centrifugation for 10 min to separate the solid part. The supernatant with water was dehydrated and WSI was determined as the mass ratio of the dehydrated material with respect to the initial mass. SC was defined as the final volume (mL) occupied by pectins (150 mg) in 10 mL beakers, after mixing with water for 2h and decanting for 12 h.

Emulsifying properties were evaluated according to the procedure described by Xu, Martinez, Yang and Guo [21] with some modifications. For emulsifying activity (EA), 5 mL of pectin solution 1.0% (*w*/*v*) was homogenized with 5 mL of soy oil in a centrifuge tube (15 mL) using a ultrasonic reactor (EcoSonics, 500 W, 20 kHz) for 1 min, and the mixture was centrifuged at 3500 rpm for 10 min. The EA was calculated by Equation 3:

EA (%) = emulsion layer volume/total volume × 100(3)

The emulsion stability (ES) was determined by heating the emulsion at 80 °C in a water bath for 30 min, followed by cooling to room temperature and centrifugation at 3500 rpm for 10 min. Finally, the ES was calculated as follows:

ES (%) = Remaining emulsion layer volume/total volume × 100(4)

### 2.4. Statistical Analysis

Except for neutral monosaccharides, all experiments were carried out in triplicate. The data were expressed as mean ± standard deviation and their normality was verified by the Shapiro–Wilk method. Significant differences were investigated by the Kruskal–Wallis non-parametric test (L*, SC and EA) and by ANOVA and Tukey tests for the other evaluated parameters with 95% confidence (*p* < 0.05) using IBM SPSS Statistics software, version 19.

## 3. Results and Discussion

### 3.1. Pectin Extraction

Images of the produced pectins can be seen in Figure 1. The yields (Table 1) were higher (~22%) for the combined extraction methods (ultrasound + microwave + enzymes, methods 2 and 3) in comparison to enzyme-free extraction (~18%, methods 1 and 4). Therefore, both cellulase and hemicellulase enzymes contributed to a small but significant increase in the yield of pectins from jabuticaba peel, with no statistical difference in relation to the used enzyme. Enzymes are able to catalyze the hydrolysis of the cell wall polymers such as cellulose and hemicelluloses with a high level of selectivity, increasing cell permeability, and thus providing an increase in the quantity of extracted pectin [5,13].

Encalada et al. [14] also reported an increase in yield with the use of enzymes to extract pectin from discarded carrots. They reported that the use of cellulase provided a significant improvement in the extraction process, with yields increasing from 6.9 to 12.4%. Adding ultrasound (US) to the extraction step provided further improvements, but the synergistic effect of enzyme extraction was only observed when using hemicellulase, which provided a significant increase in yield (20.6 to 27.1%). In our study with jabuticaba peel flours, the mechanical disorganization of the cellulose–hemicellulose network provided by the US extraction was effective for both the employed enzymes, cellulase and hemicellulase.

### 3.2. Pectin Characterization

#### 3.2.1. FTIR Analysis and DM

**Figure 2:**
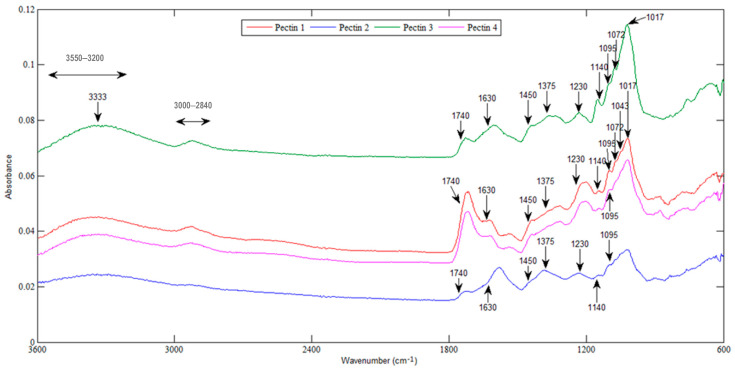
FTIR spectra of the pectins obtained from jabuticaba peel flour. Extraction methods 1 (red), 2 (blue), 3 (green) and 4 (pink).

The FTIR spectra of pectins from jabuticaba peel flour (Figure 2) are similar to the spectra of pectins from okra pods [21] and from hawthorn [22]. Regardless of the employed extraction method, similarities are observed among all pectin spectra.

A broader and stronger band between 3550 cm^−1^ and 3200 cm^−1^, centered at 3333 cm^−1^, is attributed to hydrogen bonding between –OH (hydroxyl) groups. A weak absorption band at 3000–2840 cm^−1^ arises from C–H stretching vibrations of the CH, CH_2_ and CH_3_ groups of the methyl esters, whereas the C–H asymmetrical and symmetrical bending vibrations of the methyl groups occur near 1450 cm^−1^ and 1375 cm^−1^, respectively. The band at 1230 cm^−1^ is attributed to –CH_3_CO stretching. The bands between 1200 and 900 cm^−1^ correspond to the fingerprint region, which is characteristic of each sample and may indicate variations in the composition of neutral monosaccharides, as the vibrations present in these compounds occur in this region of the spectrum [23]. The signals in the range 950–1250 cm^−1^ have been previously reported to be related to C–O–C glycoside bonds presented in the pyranose ring [24]. The peaks at 1140, 1095, 1072, 1043 and 1017 cm^−1^ were also reported for samples of commercial high methoxyl citrus pectins and purified hawthorn pectins [21,22]. Carboxyl group stretch signals that are observed for jabuticaba peel pectins were previously reported for pectins extracted from mango peels [25], namely: C–O stretching at 1140 cm^−^1, C–H stretching at 1070 cm^−1^, C–C stretching at 1018 cm^−1^ and the indication of antisymmetric and symmetric stretching of ionic carboxyl groups from 1440 to 1220 cm^−1^.

The band at 1740 cm^−1^ is attributed to the C=O stretching vibration of methyl-esterified carboxyl groups, and the one at 1630 cm^−1^ results from carboxylate group (COO–) stretching. The proportion of the area of the peak at 1740 cm^−1^ to the sum of the 1740 cm^−1^ and 1630 cm^−1^ peaks areas (corresponding to the total carboxyl groups) can be used to predict DM [12,17]. Observing these two peaks, it is noted that the pectins obtained using enzymes (blue and green spectra) seem to be of low DM and the pectins obtained only by US and heating (pink spectrum) are of high DM. In addition, it can also be observed that the band in the region 3000–2840 cm^−1^, which includes C–H stretching vibrations of methyl groups, was less intense for pectin extracted with cellulase (blue spectrum), which presented the lowest DM value (Table 1).

Regardless of the variations previously pointed out with respect to the extraction methods, the DM values obtained for jabuticaba peel flours, ranging from 44.65% to 53.01%, are high in comparison to pectins extracted from sweet lemon peel (1.2–35.1%), kinnow peel (37.17%), okra pods (39.5–43.6%) and persimmon peel (41.35%) [21,26,27,28]. DM values of pectins from jabuticaba peel flours are, however, smaller than those reported for pectins extracted from hawthorn berries (61 and 64%), lime peel without (78.4–82.2 %) and with enzymes (67.3 and 73.9%) and pomelo peel (59.85–67.01%) [12,15,22]. In the latter example, extraction was performed by MW, US and their combinations, with the lower DM obtained for the combination of both techniques [12]. A relationship between US extraction and low DM values was also observed by Encalada et al. [14] in pectins from discarded carrots (37.2 vs. 48.0%), with even lower DM values with the use of UEAE (24.0 and 27.0%). Although Chan et al. [3] reported that long extraction times at low temperature favor the de-esterification of pectin, no statistical differences were observed between the DM of pectins extracted by 5 h of heating in a water bath (method 4) and for 3 min in a MW reactor (method 1) (Table 1). These results are in agreement with the study of Dranca et al. [29], who obtained HMP from apple pomace with similar DM using MW or heating in a water bath.

Previous studies have shown that the production of LMP is also related to pH values. Encalada et al. [14] obtained LMP from discarded carrots with and without enzymes at a pH of 5.2. Ghoshal and Negi [27] produced LMP (DM of 37.17%) by hot water extraction of kinnow bark at pH 5. These results suggest that less acidic pH values may have also contributed to the extraction of LMP from jabuticaba peel flour, regardless of the use of enzymes. This is attributed to the increased activity of the pectin methyl esterase enzyme (which acts in de-methyl esterification) at pH values close to neutral, and its inhibition in high citric acid concentrations [30].

DM influences the properties of pectins and consequently their technological application. HMPs form gels in high concentrations of sugar (55–75%) and acidic systems (pH 2.5–3.5), while LMPs form gels in the presence of divalent ions, in a broader pH range (2–6), with or without a small amount of sugar. HMPs are used by the food industry as gelling agents, emulsifiers, thickeners and stabilizers in the production of jellies and for obtaining LMP by de-esterification, whereas LMPs can be used as fat substitutes in ice cream and fruit preparation for yogurt, for the formation of gels in sucrose-free products, in edible packaging and for encapsulation [3,5,13].

#### 3.2.2. Neutral Monosaccharides

**Figure 3:**
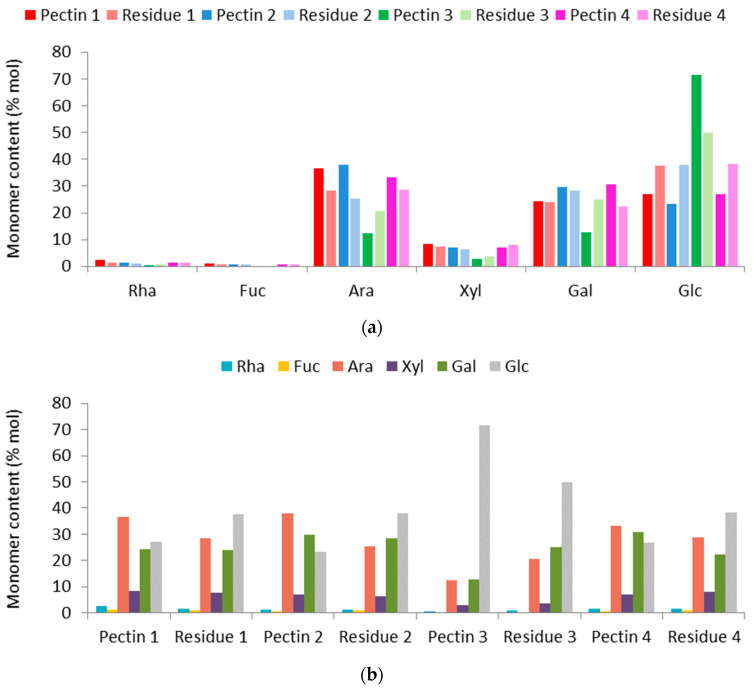
Distribution of neutral monosaccharides; pectins in darker tones and residues in lighter tones (**a**). Composition of neutral pectin sugars and extraction residues (**b**).

The neutral sugar profile (Figure 3a) shows that different monosaccharides were identified in the pectins and arabinose (Ara) was found in greater amounts than what was left in the residues obtained from methods 1, 2 and 4 (Figure 3b).

The analysis of relative monosaccharide composition revealed that arabinose (Ara) is the main compound present in pectins obtained from methods 1, 2 and 4, namely pectins 1, 2 and 4, followed by galactose (Gal) in 2, 3 and 4. The pectin obtained by hemicellulose extraction showed a higher glucose (Glc) content, which was the second most present monosaccharide in pectin 1 and the third in pectins 2 and 4. It is noteworthy to point out that Glu can come from the hydrolyzed hemicelluloses by the enzyme hemicellulase, as well as from the cellulase side activity of the hemicellulase, which hydrolyzed cellulose. Xylose (Xyl), rhamnose (Rha) and fucose (Fuc) appeared in sequence in all the produced pectins, except for in pectin 3, where there was an absence of Fuc (Figure 3b). Actually, in pectin 3, the main sugar was Clc, followed by Gal and Ara. Similar profiles were described for hawthorn and citrus pectins [22]. Commercial high methoxyl citrus pectin also showed higher content of Ara followed by Gal, but with a greater amount of Rha in relation to Glc and Xyl. Unpurified hawthorn pectins showed expressive Glc content, which was greatly reduced after purification, with a predominance of Gal followed by Ara and Rha [22]. Pectins obtained from mandarin peel by a using a conventional acid extraction method (citric acid solution, pH 1.4, 85 °C for 70 min) showed higher content of Gal followed by Ara and Rha, and smaller amounts of Glc, Xyl, mannose, fructose and Fuc [24].

The lower proportion of Rha with respect to other monosaccharides in the jabuticaba peel pectins may have been due to the use of low extraction temperatures. Chen et al. [11] compared extraction of pectins from citrus unshiu at different temperatures (25, 40 and 85 °C), and quantified lower levels of Rha and Gal in samples extracted at lower temperatures. In the present study, the fact that Gal contents are similar to Ara may be due to the greater amount of that monosaccharide in the jabuticaba peels. Miranda et al. [8] extracted a heteropolysaccharide from jabuticaba peels by hot acid and identified Gal as the prevailing monosaccharide (67.21%).

Chen et al. [11] also observed significant Ara content in pectins extracted at low temperatures, this being attributed to the hydrolysis of arabinogalactans from the extracted side chains of the polysaccharides. Chen et al. [11] reported the easiness of the hydrolysis of arabinogalactans by hot acid, with smaller amounts of Ara in the extraction residues obtained at high temperatures. Ara was also identified in residues from pectin extraction from jabuticaba peels at low temperature, suggesting that some of the pectins remained in the residues. The proportion of Ara was lower among the residues from the extraction methods that had the highest yield (2 and 3), indicating that the enzymes were efficient at hydrolyzing non-pectic compounds.

Glucose was the main monosaccharide found in the residues, indicating that cellulose may have remained in the extraction residues. The pectin extracted by cellulase had the lowest Glc content. However, a large amount of glucose was identified in the pectin extracted by hemicellulase. Part of the remaining Glc could be attributed to hemicellulase having a side effect of cellulase activity. Nonetheless, a further step in purifying this pectin may be necessary [22]. Xylose was also identified in the residues, indicating the presence of hemicellulose in the pectin extraction residues. The pectin extracted by hemicellulase presented the lowest Xyl amount. Although the xylose present in pectins may be from non-pectin polysaccharides, Xyl is also a constituent of pectins. Chan et al. [3] cite that GalA units in homogalacturonan may be replaced with residues of xylose at the C-2 or C-3 positions, generating xylogalacturonan. Xylose can also be present in side chains of RG-II (formed by Rha and GalA), as well as other monosaccharides identified in this study, such as galactose, arabinose (predominantly from galactans, arabinans and/or arabinogalactans), glucose and fucose. Analytical tools such as matrix-assisted laser desorption/ionization mass spectrometry, Fourier transform-Raman spectroscopy, carbohydrate gel electrophoresis, capillary electrophoresis and others could provide further information about the fine structures of the pectin macromolecules [3,31]. Nonetheless, the monosaccharide profile gives an indication of the compositional features of the obtained pectins, which can affect their functional properties [3,11,31].

#### 3.2.3. Phenolic Compounds

Pectins interact with polyphenols, forming complexes in which phenolic beneficial activities are preserved and also presenting beneficial synergistic effects to the digestive tract [32]. C3G and ellagic acid were the main phenolic compounds found in jabuticaba waste [33]. Therefore, they were investigated in pectins from jabuticaba peel (see Table 1).

The C3G contents ranged from 104.31 to 176.77 mg/100g in the extracted pectins, compared to 690.65 ± 40.10 mg/100g in the jabuticaba flours. These values were significantly higher than those reported for grape pomace flours (0.5 to 7.2 mg/kg) [34]. The samples obtained with enzyme free methods (pH 1.8) showed higher levels in comparison to the samples extracted at higher pH because of the use of enzymes. It is known that the stability of anthocyanins and their binding with pectic polysaccharides depends on the pH. In acidic solutions, the equilibrium form of anthocyanins flavylium cation may interact with deprotonated free carboxyl groups of the pectins so, the more free carboxylic groups available for hydrogen bonding and electrostatic interactions, the stronger the bond will be. Therefore, LMP has a greater ability to interact with anthocyanins than HMP, as well as with C3G [31,35]. In the present study, the small variation in DM between the samples did not allow for this observation; however, the obtained HMP had DM values that were not too high (<70%) (Table 1), thus allowing the interaction between pectins and C3G.

The ellagic acid levels of pectins ranged from 30.80 to 40.31 mg/100 g, without statistical differences among the samples (*p* < 0.05), compared to 202.00 ± 31.06 mg/100g in the jabuticaba flours. The values obtained for pectin are higher than those found in camu-camu (*Myrciaria dubia*) pulp powder (5.60 mg/100 g) but smaller than those reported for camu-camu waste flours (76.49 mg/100 g) [36]. Inada et al. [33] observed that only 42% of the total ellagic acid present in jabuticaba peel and seed powder was bioaccessible after gastric digestion, given the high proportion of these compounds covalently bound to the cell walls. However, the authors reported an increase of 74% in the release of ellagic acid after intestinal digestion due to the release and depolymerization of ellagitannins conjugated to the food matrix. After gut fermentation, the final bioaccessibility of ellagic acid was 30%, indicating its metabolization by gut microbiota to high lipophilicity metabolites more prone to absorption in the colon [33].

These researchers also reported a decrease in C3G bioaccessibility of jabuticaba waste after gastric (39% initial) and intestinal (19% initial) digestion and gut fermentation (≤7%), confirming its metabolization. The authors pointed out, however, that metabolites of phenolic compounds also have biological activity and may be responsible for the beneficial effects of jabuticaba [33]. Whether by the absorption of these phenolics or their metabolites, the presence of C3G and ellagic acid in pectins is interesting given the already reported health benefits of these compounds, such as anti-inflammatory effects [37,38] and effectiveness for the prevention or improvement of type 2 diabetes mellitus [39,40]. Further studies are needed in order to access the bioavailability of these compounds in the extracted pectins.

#### 3.2.4. Technological Properties

Images of the pectins obtained from jabuticaba peel flours are displayed in Figure 1 and their color parameters in Table 2. Luminosity (L*) values ranged from 13.08 to 20.59%, although there was no statistical difference among the samples (*p* < 0.05). The jabuticaba peel pectins are darker than pectins obtained from discarded carrots by UAE + hemicellulase (L* = 40.6%) and UAE + cellulase (L* = 47.2%) [14]. The low luminosity of the samples may be exploited for use in whole foods or other dark products with structure or function claims. According to the hue angle measurements (4.04–35.62), the extracted pectins presented a reddish tone, characteristic of the C3G flavylium cation [35], with a difference in tone depending on the extraction pH (tendency to be orange at higher pH, methods 2 and 3). In relation to color intensity (c*), the pectin extracted by cellulase was the least pigmented, and the sample extracted by water bath was the most pigmented, without statistical difference with respect to methods 1 and 3 (*p* < 0.05). Nonetheless, both variations in color intensity and tone cannot be directly visualized because of the low luminosity values.

Results obtained for the technological properties of the extracted pectins and commercial citrus pectin (CCP) can be seen in Table 3. Pectins from jabuticaba peels and CCP showed no differences in WRC and ORC, and the values for both properties were around 1.0 g/g. WRC was low in comparison to pectin-rich dietary fiber from citrus peel (8.32 g/g) [41], but the time that the fibers were exposed to water was much longer than the time for the citrus samples (12 h vs. 30 min in this study). WRC values were closer to those of pectins from heat-treated alperujo (2.18 g/g) that were subjected to 5 h of contact with water [42] and slightly higher than LMP from okra pods (0.82 g/g) [21]. WRC represents the amount of water held by pectins, and is a factor that affects food texture and depends on different factors, including molecular weight [21]. Xu et al. [21] observed the degradation of pectin molecules (decrease in molecular weight) when the raw material was dried above 20 °C, with consequent decrease in WRC. In our study, the peels were dried at 60 °C; therefore, freeze-drying of jabuticaba peels could improve this property. The time of exposure of the fibers to water also seems to affect WRC and needs to be further investigated.

Oil retention capacity values were similar to pectins from alperujo (1.48 g/g) [42], and slightly lower than pectin-rich fibers from citrus peel (2.12 g/g) [41] and pectins from okra pods (2.84 g/g) [21]. ORC represents the amount of oil absorbed by pectins, affecting sensory properties.

Swelling capacity ranged from 7.00 to 15.11 mL/g, comparable to pectin-rich fibers from citrus peel (8.08 mL/g) [41]. The data did not follow the normal distribution and the non-parametric test indicated statistical difference between the extreme values. However, when analyzing the absolute values, it can be noticed that the pectins obtained by UMEAE had a higher SC value. SC may vary depending on pectin content, particle size and surface area [41]. Li, He, Lv and He [43] observed that MAE, EAE and UAE contributed to an increase in SC (6.5, 7.2, and 7.3 mL/g, respectively) of water-soluble dietary fiber from apple pomace, in comparison to a conventional acid extraction method (2.5 mL/g). The authors argued that the effects of cavitation and intramolecular heating on the cell wall disruption by US and MS, respectively, made the structure of extracted fibers bulky and porous, allowing water to flow more easily into the fiber’s interspace. They further discussed that cellulase may effectively destruct the cell wall by disrupting the polysaccharide chains linked β(1–4), leading to a reduction in particles size and an increase in the surface area of the extracted fibers, providing greater water retention and exposure of the more hydrophilic groups to the water bond [43]. The pectin extracted by hemicellulase (method 3) presented higher water solubility (WSI = 3.74 g/100g), and this value was significantly higher (*p* < 0.05) than the commercial pectin sample (WSI = 2.01 g/100g).

**Figure 4:**
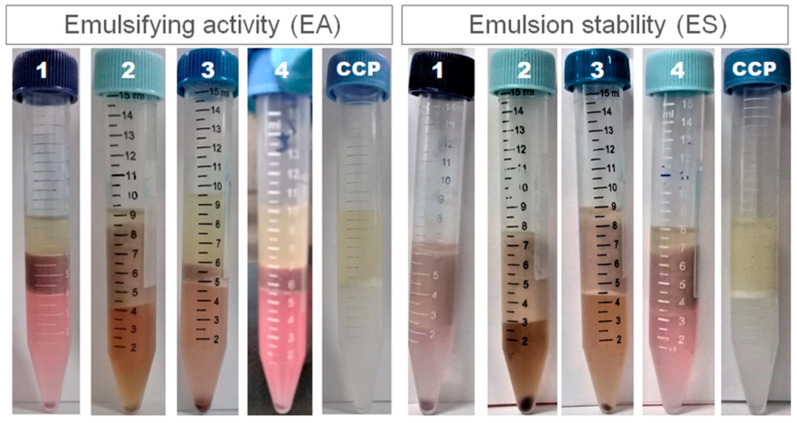
Emulsifying properties. Images of one of the replicates from each experiment.

The results obtained for the emulsifying properties of pectins, emulsifying activity (EA) and emulsion stability (ES), are also displayed in Table 3 and images are shown in Figure 4. The commercial pectin sample (CCP) presented the lowest values for emulsifying properties, with statistical difference in comparison to the pectin extracted by cellulase (method 2). This can be related to the higher DM value of CCP in comparison to the pectins herein produced. Pectins extracted from persimmon peel and hawthorn also showed better emulsification performance than commercial citrus pectins [22,28]. Among the pectins obtained from jabuticaba peel flour, EA ranged from 10.58 to 48.77%, and ES ranged from 43.30 to 55.05%. EA from the pectin extracted by cellulase was slightly above the value reported for LMP from oven dried okra pods (42.5%), and ES values from all the jabuticaba peel pectins were much higher [21]. Unlike the study by Xu et al. [21], who extracted pectins from okra pods by the conventional method of heating at high temperature, all samples in the present study improved their emulsification performance during the ES tests which aimed to induce coalescence and destabilize the emulsions under high temperature conditions. Given this performance, pectins from jabuticaba peel could be further tested as emulsifiers from natural sources in products such as ice cream, sauces, breads and cakes.

Roman et al. [22] and Xu et al. [21] commented that a combination of factors such as high protein content, higher proportion of HG in relation to RG-I, lower DM (for providing electrostatic stabilization), degree of acetylation and higher molecular weight of pectic polysaccharides might improve the emulsifying properties. Consistent with this analysis, the sample of jabuticaba peel pectin extracted by cellulase showed lower DM and higher EA and ES. However, some of the factors that affect emulsifying properties, including protein content, HG/RG-I ratio and molecular weight, were not investigated in the present study.

Jiang et al. [28] reported that tannins can also improve emulsifying performance. The authors commented that phenolic acids can bind to arabinose and galactose by ester linkages, and the carboxyl group of the pectins can be cross-linked intermolecularly with the hydroxyl group of the phenolics by hydrogen bonding and hydrophobic interactions, forming a heterogeneous and aggregated network in the continuous phase that improves the emulsifying properties [28]. In an experiment with pectins, Shuai et al. [44] observed higher emulsion stability in samples with higher protein and ferulic acid contents. The authors explained that the proteins and the ferulic acid act as a kind of “bridge” between the oil and polysaccharide phases, which allows the pectin molecules to act as anchors attached to the water–oil interface, while the carbohydrate moiety forms a moisturizing layer, preventing the aggregation or binding of the emulsion droplets [44]. Cho, Yu and Hwang [45] observed that the incorporation of ellagic acid into sodium caseinate–HMP emulsions improved oxidative stability by reducing lipid hydroperoxides. Ellagic acid is poorly soluble in water; however, due to its acidic nature, it dissolves better in a basic solvent [45]. Despite the fact that ellagic acid content (Table 1) in the extracted pectins was not statistically different (*p* > 0.05), the pectin extracted by cellulase (at less acidic pH) showed slightly more ellagic acid. Nonetheless, emulsion stability cannot be solely explained by the ellagic acid content. A combination of the previously mentioned factors may have contributed to the emulsifying properties observed in the prepared jabuticaba peel pectins.

## 4. Conclusions

In this study, flours prepared from jabuticaba peel were investigated as a potential source of pectin. A comparative evaluation of extraction methods was presented. All methods were based on US extraction followed by microwave heating (method 1) including enzymatic treatments employing cellulase (method 2) or hemicellulase (method 3) or followed by conventional heating in a water bath (method 4). Pectin yields ranged from approximately 18% to 22%, and were slightly higher for the methods that employed enzymes. Methods that did not employ enzymes resulted in high methoxyl pectins (53 % DM), as opposed to low methoxyl pectins (44 % and 48.6 % DM) obtained after enzyme treatment. C3G and ellagic acid were the main phenolic compounds found in jabuticaba peel flour, with the higher C3G levels obtained with enzyme-free extraction. All the prepared pectins presented a dark reddish tone, good emulsifying properties and high swelling capacity in comparison to the commercially available pectin. In this study we evaluated for the first time the potential of combining ultrasound, microwave and enzymes for pectin extraction, resulting in improvements in the emulsifying performance.

## Figures and Tables

**Table 1:** Yield, degree of methyl-esterification (DM) and phenolic contents of the pectins obtained from jabuticaba peel using different extraction methods.

Method*	Yield (%)	DM (%)	Phenolic Compounds	
			Cyanidin-3-O-Glucoside (mg/100g)	Ellagic Acid (mg/100g)
1	17.79 ± 0.63b	52.93± 0.71a	176.77 ± 5.97a	33.74 ± 2.85a
2	21.80 ± 2.25a	44.65 ± 2.02c	104.31 ± 17.57b	40.31 ± 9.05a
3	22.06 ± 0.42a	48.58 ± 0.21b	115.94 ± 18.13b	24.85 ± 5.17a
4	18.38 ± 0.35b	53.01 ± 0.87a	169.73 ± 13.93a	30.80 ± 4.55a

*1 = UMAE; 2 = UMAE + cellulase; 3 = UMAE + hemicellulase; 4 = US + water bath. Mean ± standard deviation (n = 3). Different letters in the same column indicate that values are significantly different (*p* < 0.05). DM = degree of methyl-esterification.

**Table 2:** Color parameters of the pectins obtained from jabuticaba peel using different extraction methods.

Method**	^1^ L* (%)	h	c*
1	13.08 ± 0.26a	4.04 ± 2.33b	4.89 ± 0.77a
2	16.73 ± 1.71a	35.62 ± 9.20a	3.14 ± 0.23b
3	20.59 ± 2.28a	25.51 ± 5.01a	4.67 ± 0.31a
4	13.18 ± 0.33a	6.20 ± 0.67b	5.52 ± 0.25a

**1 = UMAE; 2 = UMAE + cellulase; 3 = UMAE + hemicellulase; 4 = US + water bath. Mean ± standard deviation (n = 3). Different letters in the same column indicate that values are significantly different (*p* < 0.05). ^1^ Kruskal–Wallis non-parametric test, ANOVA and Tuckey tests for the other parameters. L* = luminosity; h =hue angle; c* = chroma.

**Table 3:** Technological properties of the pectins obtained from jabuticaba peel using different extraction methods.

Method*	Technological Properties				Emulsifying Properties	
	ORC (g/g)	WRC (g/g)	^1^ SC (mL/g)	WSI (g/100g)	^1^ EA (%)	ES (%)
1	1.02 ± 0.00a	1.03 ± 0.01a	7.00 ± 0.33b	2.22 ± 0.66ab	20.88 ± 3.76ab	49.99 ± 6.20ab
2	1.02 ± 0.00a	1.03 ± 0.02a	9.78 ± 0.77ab	3.21 ± 0.49ab	48.77 ± 6.73a	55.05 ± 1.52a
3	1.04 ± 0.01a	1.02 ± 0.01a	15.11 ± 1.39a	3.74 ± 0.40a	10.58 ± 3.29ab	43.30 ± 3.26ab
4	1.03 ± 0.01a	1.02 ± 0.03a	7.33 ± 0.67ab	2.27 ± 0.72ab	13.41 ± 4.27ab	46.88 ± 5.41ab
CCP**	1.02 ± 0.01a	1.04 ± 0.03a	-	2.01 ± 0.54b	5.57 ± 0.31b	40.63 ± 4.27b

*1 = UMAE; 2 = UMAE + cellulase; 3 = UMAE + hemicellulase; 4 = US + water bath. **Commercial citrus pectin. Mean ± standard deviation (n = 3). Different letters in the same column indicate that values are significantly different (*p* < 0.05). ^1^ Kruskal–Wallis non-parametric test, ANOVA and Tuckey tests for the other parameters. EA = emulsifying activity; ES = emulsion stability; ORC = oil retention capacity; WRC = water retention capacity; WSI = water solubility index; SC = swelling capacity.

## Data Availability

Data is contained within the article.
